# Integrated Organotypic Slice Cultures and RT-QuIC (OSCAR) Assay: Implications for Translational Discovery in Protein Misfolding Diseases

**DOI:** 10.1038/srep43155

**Published:** 2017-02-24

**Authors:** Naveen Kondru, Sireesha Manne, Justin Greenlee, Heather West Greenlee, Vellareddy Anantharam, Patrick Halbur, Arthi Kanthasamy, Anumantha Kanthasamy

**Affiliations:** 1 Department of Biomedical Sciences, College of Veterinary Medicine, Iowa State University, Ames, IA 50011, USA; 2 Virus and Prion Research Unit, National Animal Disease Center, Agricultural Research Service, United States Department of Agriculture, Ames, IA 50011, USA; 3 Veterinary Diagnostic and Production Animal Medicine, College of Veterinary Medicine, Iowa State University, Ames, IA 50011, USA.

## Abstract

Protein misfolding is a key pathological event in neurodegenerative diseases like prion diseases, synucleinopathies, and tauopathies that are collectively termed protein misfolding disorders. Prions are a prototypic model to study protein aggregation biology and therapeutic development. Attempts to develop anti-prion therapeutics have been impeded by the lack of screening models that faithfully replicate prion diseases and the lack of rapid, sensitive biological screening systems. Therefore, a sensitive model encompassing prion replication and neurotoxicity would be indispensable to the pursuit of intervention strategies. We present an ultra-sensitive screening system coupled to an *ex vivo* prion organotypic slice culture model to rapidly advance rationale-based high-throughput therapeutic strategies. This hybrid **O**rganotypic **S**lice **C**ulture **A**ssay coupled with **R**T-QuIC (OSCAR) permits sensitive, specific and quantitative detection of prions from an infectious slice culture model on a reduced time scale. We demonstrate that the anti-prion activity of test compounds can be readily resolved based on the power and kinetics of seeding activity in the OSCAR screening platform and that the prions generated in slice cultures are biologically active. Collectively, our results imply that OSCAR is a robust model of prion diseases that offers a promising platform for understanding prion proteinopathies and advancing anti-prion therapeutics.

Prion diseases, or transmissible spongiform encephalopathies (TSEs), are chronic, lethal neurodegenerative disorders affecting both humans and animals, and a cure for these devastating brain diseases has yet to be identified. The common prion diseases of animals include bovine spongiform encephalopathy (BSE) in cattle, scrapie in sheep and goats, and chronic wasting disease (CWD) in cervids. Major human prion diseases are Creutzfeldt-Jakob disease (CJD), Gerstmann–Sträussler–Scheinker syndrome (GSS) and fatal familial insomnia (FFI)^1^. Prion diseases are often hard to detect due to their long incubation periods as well as clinical presentations that overlap with other neurological disorders. In addition to classical prion diseases, emerging evidence suggests that other protein misfolding disorders (PMDs) like Parkinson’s disease (PD) and multiple systems atrophy (MSA) have misfolded α-synuclein proteins that are experimentally transmissible^2^,^3^ and other PMDs like Alzheimer’s, FTDs, Huntington’s *etc.,* also have unique amyloids capable of prion-like aggregation and experimental propagation^4^,^5^. The normal cellular form of prion protein (PrP^C^) is richly distributed throughout the nervous system and lymphoid tissues. PrP^C^ is known to play a role in oxidative stress, apoptotic signaling, and other biological functions including interactions with metals^6^,^7^,^8^. Although the mechanisms underlying the templated conversion of PrP^C^ into its misfolded isomer, PrP^Sc^ (*Sc* denotes scrapie; also referred to as PrP^res^ for protease-resistance), remains poorly understood, PrP^Sc^ associated with TSEs are oligomers, fibrils or aggregates that contribute to neuropathological processes^9^.

Detection of misfolded prion in a high-throughput format is necessary for rational therapeutic designs^10^,^11^. Currently, mouse bioassays serve as a predominant method of assessment for prion infectivity. However, these models present several obstacles to developing high-throughput assays. For instance, incubation times can extend to excessively long durations and clinical signs are typically not manifested until the terminal stages of infection^6^. Furthermore, working with infected animals raises concerns about occupational safety and the increasing costs associated with care and management of laboratory animals^12^,^13^. Efforts to establish *in vitro* models of prion diseases are often met with limited success due to the inability of cells to maintain persistent infectivity over time. Results generated from one or two cell models has not readily translated to other preclinical and clinical models of different prion strains of animals and human prions^14^,^15^. Notably, *in vitro* cell models fail to recapitulate the neuropathological features of prion disease. Thus, a suitable alternative to chronically infected animal models could help advance prion research via rapid validation of therapeutic options.

The recently developed real-time quaking-induced conversion (RT-QuIC) assay for prions is gaining wide acceptance for its ultra-sensitive detection of prions from a variety of samples and has the potential to become a rapid and sensitive prion detection assay. This robust and reproducible, high-throughput prion detection assay can be adapted for both animal and human prion diseases, and it is compatible with a wide range of prion strains, allowing strain discrimination^16^,^17^,^18^,^19^,^20^. Notably, RT-QuIC was shown to detect prions with a million-fold greater sensitivity than had been achieved via the immunoblot detection of scrapie prions^21^ and also with superior specificity, stability, and reproducibility^17^ for diagnostic screening. This assay also was adopted for quantitative and qualitative estimation of prion titers in samples using endpoint titration of prions defined by seeding activity^22^,^23^. Thus, we postulated that integrating the **O**rganotypic **S**lice **C**ulture **A**ssay with **R**T-QuIC (OSCAR) would prove to be the ideal platform for fulfilling a long-standing gap in the translational arena of prion diseases. Herein, we show that OSCAR is a rapid, specific and quantitative method that can be readily adopted for translational research and for understanding the molecular mechanisms underlying protein misfolding disease processes.

## Results

### *Ex vivo* cerebellar slice cultures exhibit hallmarks of prion infection and neurodegeneration

**Figure 1:**
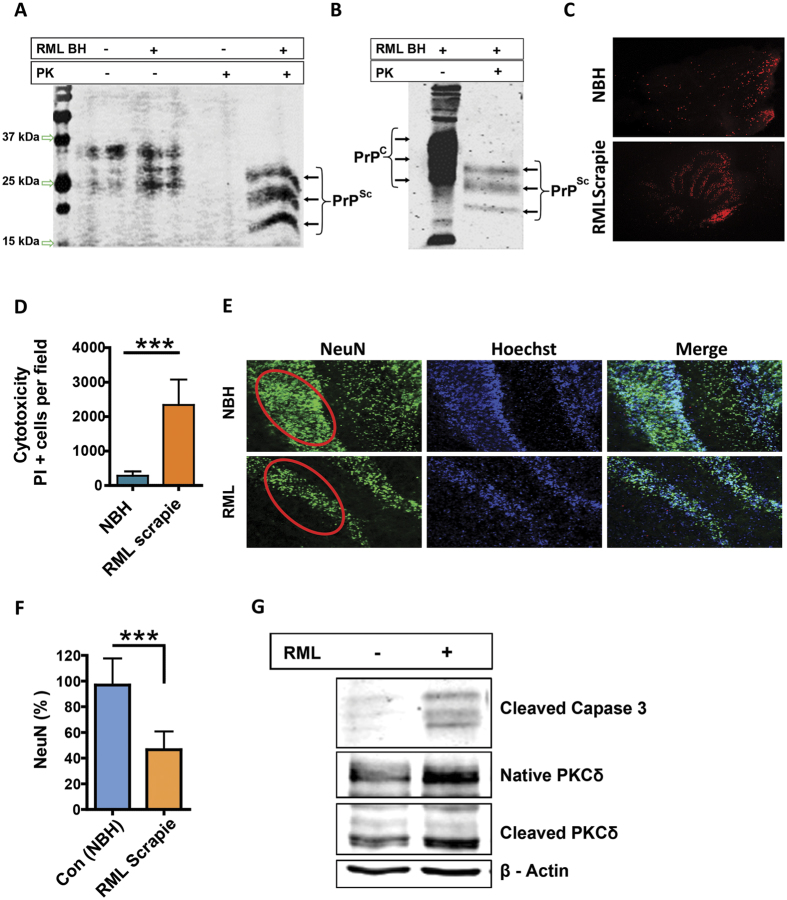
Prion-infected organotypic slice cultures exhibit distinct biochemical and neurodegenerative changes. (**A**) Lysates from organotypic cerebellar slices exposed to NBH or RML scrapie and cultured for 35 days were subjected to PK treatment and then Western blotted (undigested samples were loaded after diluting 10-fold to prevent over-exposure on blots). RML-treated slices accumulate the PK-resistant PrP signal, but NBH-treated slices did not. (**B**) An equal amount of brain homogenates from WT mice was infected with RML scrapie either undigested or digested with proteinase K (PK) to detect PK resistance (designated as PrP^Sc^) on immunoblot. Membranes were probed with anti-PrP (α-helix 1) mouse monoclonal antibody, POM1. The banding profile showed differential migration with banding profiles consistent with PrP^C^ for undigested and PrP^Sc^ for digested samples. (**C**,**D**) Propidium Iodide (PI) incorporation to visualize dead cells in 49-dpi slice cultures. Quantification of PI^+^ cells from NBH or RML scrapie-infected slices using ImageJ. RML scrapie-infected cerebellar slices showed significantly higher cell death when compared to NBH-treated brain slice cultures. Data were analyzed using a two-tailed t-test; n = 6 biological replicates. Data are mean ± SEM (*p < 0.05, **p < 0.01, ***P < 0.001). (**E**,**F**) Slices were stained with NeuN to determine any neuronal loss at 42 dpi. RML scrapie-infected slice cultures suffered significant neuronal loss as evidenced by NeuN^+^ area of percent NBH-treated slices. (**G**) RML scrapie-infected slice cultures show pro-apoptotic signaling as evidenced by both upregulation and proteolytic activation of PKCδ and augmented cleaved caspase 3 when compared to controls. All the samples were normalized to the loading control β-actin.

To test whether organotypic slices accumulate PK-resistant prions, the molecular hallmark of prion infection in culture, we prepared organotypic cerebellar slices from 9- to 12-day-old WT pups and maintained them as *ex vivo* slice cultures after infecting them with RML scrapie. After five weeks in culture, slices were harvested and lysates were digested with Proteinase-K for the electrophoresis profile. The slices infected with RML scrapie show an obvious pattern of di-, mono-, and non-glycosylated PK-resistant bands (PrP^Sc^) on immunoblots with bands shifted lower than those from uninfected slices (Fig. 1A). This illustrates that the resulting PK-resistant fragments have lower molecular weight when compared to the undigested protein. This banding profile is consistent with the terminally sick brain homogenates of mice (Fig. 1B), indicating the amplification of prions in culture. Next, we evaluated slice viability using propidium iodide (PI) staining. The slice cultures showed significantly less PI-positive staining during the culture periods (<5%) tested up to 9 weeks when compared to a positive control (Sup Fig. 1). However, after 42 days in culture, slices infected and cultured with RML scrapie showed a significant increase in PI-positive cells, concentrated in the granular cell layer when compared to slices treated with non-infectious brain homogenate (NBH) (Fig. 1C,D). Next, we examined the neuronal density of NeuN^+^ cells in the slices. RML scrapie-infected slices displayed a significant neuronal loss in the granular layer of the cerebellum when compared to NBH-treated slices after 42 days post inoculation (dpi) (Fig. 1E and F). Furthermore, we characterized pro-apoptotic markers in RML-infected cerebellar slice cultures. A pro-apoptotic signaling cascade in RML scrapie-infected slices was evidenced by caspase-3 activation and a significant increase in the novel protein kinase C isoform δ (PKCδ) and its cleaved fragment (Fig. 1G, full blots in Sup Fig. 5). Taken together, these results suggest that slice cultures serve as a representative model for prion replication and neurotoxicity in a culture environment while preserving the complex connections.

### RT-QuIC assay provides a versatile platform for sensitive detection of prions in *ex vivo* slice cultures

**Figure 2:**
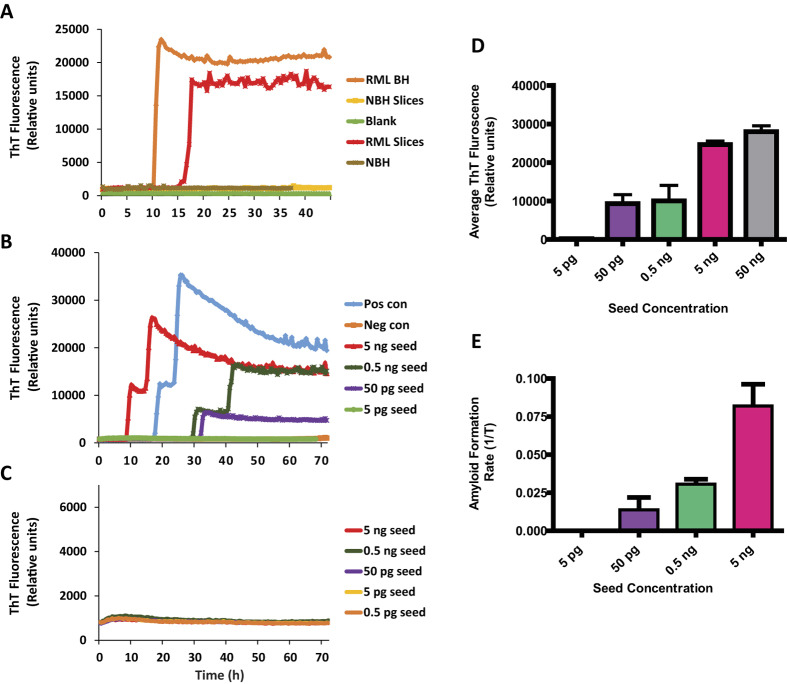
Sensitive and specific prion seeding activity from organotypic slice cultures on RT-QuIC assay. (**A**) Slice cultures from WT mice (*C57BL/6* background) efficiently seed the RT-QuIC assay. Representative RT-QuIC responses shown for the reactions seeded with 5 ng of WT homogenates from either NBH or RML scrapie-infected slice cultures (21 dpi) or brain homogenates from mock or terminally-infected scrapie mice. Uninfected samples did not show seeding activity and stayed at the baseline throughout the reaction. (**B**) RT-QuIC traces for a log dilution series from *Tga*20 slice cultures (21 DPI) showing clear concentration-dependent seeding activity. (Positive control = mock or clinical scrapie mice. Neg Con = mock/normal brain homogenate-infected samples). (**C**) Cultured slices from *PrP*^−/−^ did not show seeding activity (both NBH and RML scrapie groups) at any seed concentration. Data from the traces shown are average of three replicates for each dilution. (**D**) Quantification of average ThT fluorescence values obtained over 60-hour period for each seed concentration tested from *Tga*20 slice cultures (21 dpi) shows a seed concentration-dependent increase. (**E**) Quantification of average amyloid formation rate (AFR) for each seed concentration tested from *Tga*20 slice cultures (21 dpi) showed a seed concentration-dependent increase in AFR.

Following the establishment of mouse organotypic cerebellar slices cultured *ex vivo,* we evaluated the RT-QuIC assay for testing nanogram to femtogram quantities of misfolded prions in slice cultures. Only the scrapie-infected slices and terminally sick mouse brain homogenates (positive control) were amplified consistently across multiple experiments (n > 20), whereas NBH-treated slices or healthy mouse brain homogenates did not show seeded amplification over the course of 72 h of reaction (Fig. 2A). To confirm that this amplification occurred due to specific amplification of prions in slice cultures, we used transgenic prion mouse models to probe for prion seeding activity. We prepared slices from *Tga*20 pups that overexpress prion protein ~10 fold and/or PrP^−/−^ pups with no PrP expression, infected them with either RML scrapie or mock normal brain homogenates and cultured them for 28 days before subjecting their samples to RT-QuIC assay. Only slices from *Tga*20 mice showed seeding activity (Fig. 2B), whereas NBH-treated slices or PrP^−/−^ slices infected with RML scrapie did not show any seeding activity at any of the dilutions tested (Fig. 2C). The amplification kinetics from *Tga*20 slices were seed dose-dependent as demonstrated by the time required to reach threshold and maximum average fluorescence readings (Fig. 2D,E). Collectively, these findings suggest that true prion replication in organotypic slice cultures and the seeding activity is governed by the expression of cellular prion protein.

### Kinetics of prion augmentation in slice cultures

**Figure 3:**
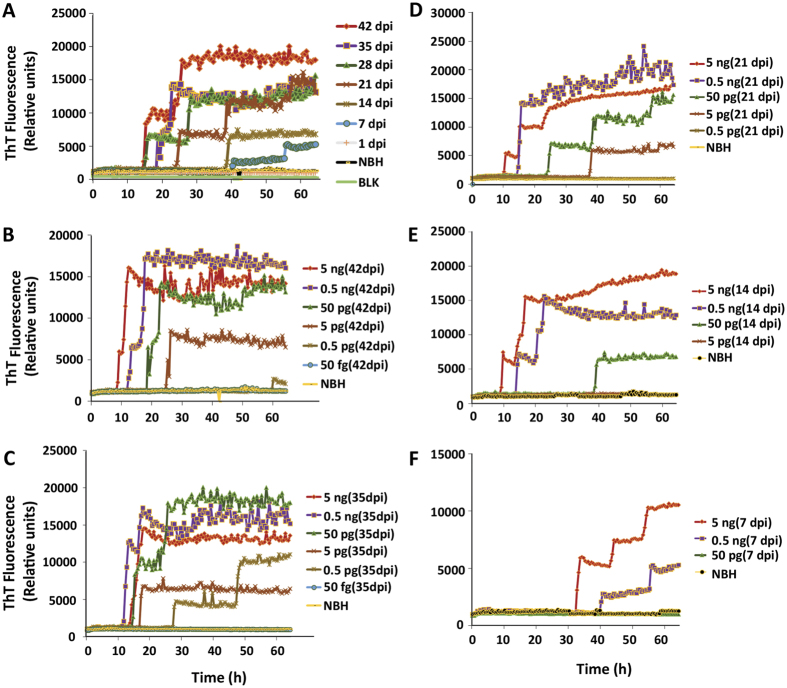
Temporal seeding activity from prion-infected organotypic slice cultures: RT-QuIC end-point dilution analysis in slice cultures from wild-type mice. (**A**) Organotypic cerebellar slices from WT mice infected with scrapie show time-dependent seeding activity. After RT-QuIC reactions were seeded with 5 ng of homogenate, seeding activity occurred only in RML scrapie-infected slice cultures, not in NBH-exposed slice cultures. NBH-treated slices and RML scrapie-infected slices at 1 dpi did not show seeding activity. The earliest detection of seeding activity was observed at 7 dpi with longer lag phases. Seeding strength showed a clear time-dependent increase as judged by time to reach threshold. Seeding activity was also noted at 14 dpi, but the traces became stronger from slices at 21 dpi and continued to increase for later time points (28, 35 and 42 dpi). Data from each trace shown are the average ThT fluorescence of three replicate wells. (**B**–**F**) Seeded amplification of prions at log dilutions tested from cultured slices. Traces shown are average of 3 replicates from 42 dpi (**B**), 35 dpi (**C**), 21 dpi (**D**), 14 dpi (**E**) and 7 dpi (**F**).

We systematically characterized the seeding activity of misfolded prions following various dose and incubation times. The seeding activity in RT-QuIC assays of samples from organotypic slice cultures became progressively stronger as culture duration increased. Cultured slices infected with NBH or RML scrapie were harvested at multiple time points ranging from 1 to 42 days dpi for RT-QuIC assay. Seeding activity was detected as early as 7 dpi (Fig. 3A). However, seeding activity increased substantially in RT-QuIC assays only after 14 dpi when PrP^Sc^ started to buildup. This is comparable with previous reports demonstrating PK-resistant material deposition only after two weeks in RML scrapie-infected slices^12^,^24^,^25^. Nonetheless, slices harvested at 21 dpi showed even stronger seeding activity, and the seeding activity in RT-QuIC assays did not peak until around 42 dpi (Fig. 3A). The slices harvested immediately after starting the culture (1 dpi) did not show discernible seeding activity, suggesting that the seeding activity did not begin from the inoculum used to infect slices. This time-dependent intensification of seeding activity in RT-QuIC assays (Fig. 3A) clearly suggests the amplification of prions in the e*x vivo* cultured slices. To further determine the seeding efficiency of prions replicated in slice cultures at various time points after infection and culture (7, 14, 21, 35 and 42 dpi), we tested serial 10-fold dilutions of slice culture homogenates (5 ng–50 fg) (Fig. 3B–F). Slices cultured for 42 and 35 dpi (Fig. 3B and C, respectively) showed greater seeding activity and had both shorter lag phases and stronger relative fluorescence (RFU) values compared to 21 dpi (Fig. 3D). The seeding activity of prion-infected slice cultures at 14 dpi and 7 dpi was not as robust as at 21 dpi (Fig. 3E,F). Additionally, the slices harvested after 7 and 14 dpi took longer to reach thresholds and the average RFU was reduced 2- to 3-fold with respect to 35-dpi cultures. Furthermore, the ThT fluorescence intensity generated from the RT-QuIC seeds was correlated (r = 0.855) with time spent in culture (Sup Fig. 2). These results clearly demonstrate that RT-QuIC captures both amount and rate of prion infection in the *ex vivo* brain culture model.

### Quantitative estimation of infectious titers from prion-infected slice cultures based on seeding activity in RT-QuIC assay

**Figure 4:**
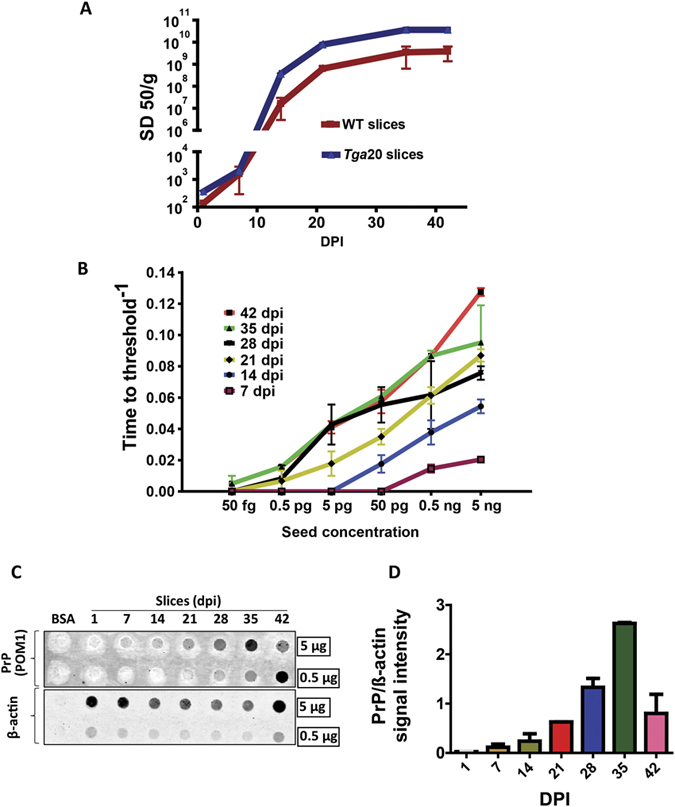
Rapid kinetics and quantitative assessment of prions from slice cultures using RT-QuIC assay. (**A**) The average SD_50_/g of protein (Spearman-Kärber estimates) of slice cultures was calculated based on endpoint dilutions from (n = 6 technical replicates) and plotted at time points (1, 7, 14, 21, 35 and 42 DPI) for both WT (n = 3 biological replicates) and *Tga*20 (n = 1 biological replicate) slice cultures. (**B**) The amyloid formation rate (AFR) was estimated by using the inverse of time in hours at which a trace crosses threshold fluorescence. The linear response AFR for cultured slices was both time- and seed concentration-dependent. (**C**) Dot-blot showing immunoreactivity against POM1 antibody. Progressive increase in immunoreactivity for PrP with respect to time spent in culture, starting at 14 dpi. Immunoreactivity against β-actin was used as a loading control for normalization. (**D**) Quantitative estimation of PrP immunoblot signal intensity.

Quantifying the seeding activity of slice cultures would help us to understand the kinetics and dynamics of prion aggregation, which would further aid in developing disease-modifying therapies or improving existing compounds. To quantify prion seeding activity, we tested endpoint seeding dilutions of slice cultures as described in previous reports using the Spearman-Kärber approach for calculating the 50% infective dose (ID_50_) in virus titration experiments^23^. We performed RT-QuIC assays on log dilutions (10 fold) of slice homogenates (as shown in Fig. 3B–F) to determine the seeding activity of different dilutions at each endpoint. The Spearman-Kärber method relies on the number of wells turning positive at each dilution point to determine arbitrary SD_50_ (seeding activity, 50%) values, representing the dilutions of slice homogenate at which 50% of the wells became positive in RT-QuIC reactions. Over a wide range of time points, SD_50s_ showed a clear pattern of increasing values with increasing culture durations for homogenates from both WT and *Tga*20 organotypic brain slices (Fig. 4A). In addition, the seeding activity for *Tga*20 slice cultures was always higher at all time points tested possibly due to a higher quantity of seeding prions. The average values (SD_50_/g) obtained by this method peaked at 35 dpi for both WT slices (6.32 × 10^9^) and for *Tga*20 slice cultures (3.64 × 10^10^). Serial dilutions of RML scrapie brain homogenates that were used to calculate SD_50_ achieved a similar seeding activity per gram^23^, suggesting that the prion titers accumulated in cultured slices were comparable to *in vivo* conditions (Sup Fig. 3). Next, we explored the threshold-crossing time (Tt), which is the time taken for the fluorescence intensity to cross the threshold, as a measure of seeding power of sample tested, as described in previously published reports, to indirectly calculate the amyloid formation rate (AFR), which is the inverse of Tt (in hours). To gain a comprehensive understanding of prion replication, we used this model to study the kinetics of amyloid formation based on Tt derived from different seeding dilutions (50 fg–5 ng) of slice culture homogenates at 7–42 dpi (Fig. 4B). Not only was AFR correlated with the dpi of cultured slices, but also higher seed concentrations were directly proportional to AFR. The lower dilutions that never turned positive in an RT-QuIC assay were predictably considered to have zero AFR. In addition to this, PrP immunoreactivity was examined semi-quantitatively using dot blot assay (as described in methods). Homogenates prepared from slices cultured 1–42 dpi (0.5 μg and 5 μg) were transferred to nitrocellulose membranes and treated with the prion antibody POM1 to visualize PrP immunoreactivity. The signal intensity of PrP, normalized to the loading control β-actin, increased in a time-dependent fashion (Fig. 4C,D). These results demonstrate that the OSCAR platform enables us to quantify the progressive increase in amyloid formation rate and determine the prion titers from cultured slices. This is advantageous when determining the effect of pharmacological modulators on amyloid formation as an indirect marker of disease progression under *in vivo* conditions.

### Effect of anti-prion compounds on seeding activity in RT-QuIC assay of prion-infected slice cultures

**Figure 5:**
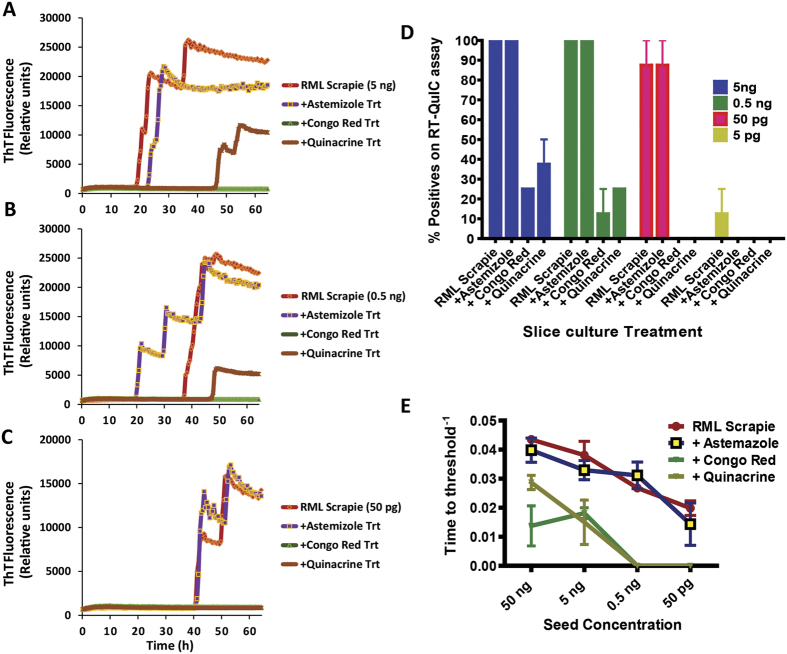
OSCAR model to determine the anti-prion activity of compounds. (**A**–**C**) Compounds known to have anti-prion activity against RML scrapie prions were tested. RT-QuIC responses shown for 3 different seed concentrations (5 ng, 0.5 ng, and 50 pg) with or without compounds. Each trace shown is an average of 4 replicates (n = 2 biological replicates). (**D**,**E**) Quantification of anti-prion activity of compounds with respect to percent ThT-positive wells at different seed dilutions. Amyloid formation rate (AFR) represents the anti-prion activity of compounds tested.

To extend the applicability of our OSCAR model, we applied some already known anti-prion compounds (astemizole, Congo red and quinacrine) to slice cultures to test their ability to influence the seeding activity of prions from RML scrapie-infected slice cultures. First, we measured the seeding ability of slice cultures tested in two independent experiments with high reproducibility (Sup Fig. 4). Next, we compared the seeding ability of the slice cultures treated with compounds at various dilutions (Fig. 5A–C). Both Congo red and quinacrine, but not astemizole, showed reduced seeding activity at 5 ng (Fig. 5A) and 0.5 ng (Fig. 5B) seed concentrations. Similarly, when a dilution of 50 pg was used as seed, both Congo red and quinacrine, but not astemizole, completely inhibited seeding activity (Fig. 5C). Quantification of percent positive wells tested for each treatment shows a clear pattern of seeding activity for the compounds (Fig. 5D). Anti-prion activity was clearly manifested by a reduction in AFR (Fig. 5E) for two of the compounds. Together, these results suggest a true reduction in seeding activity upon addition of the anti-prion compounds Congo red and quinacrine. Furthermore, these results highlight the utility of the OSCAR model for determining the preclinical efficacy of compounds that are effective against prion seeding activity.

### Serial propagation of prions and utility of non-cerebellar brain slice cultures to confirm prion infectivity

**Figure 6:**
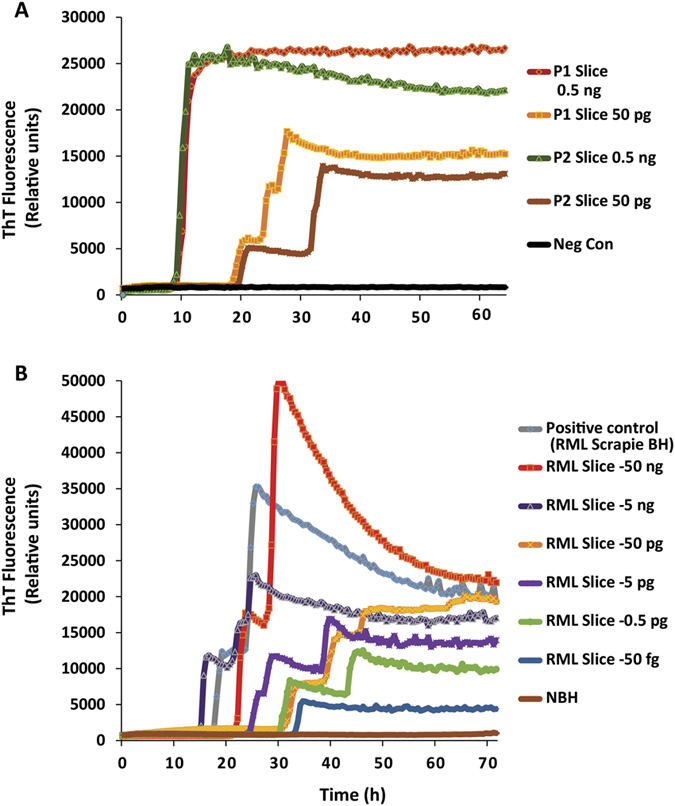
Prions generated in slice cultures are biologically active and OSCAR model can be adapted to non-cerebellar brain slice cultures. (**A**) Efficient transmission of prions in slice cultures from serial passages shows comparable seeding activity between P1 and P2 slices. Traces are average of 3 technical replicates for each dilution. Additional dilutions were tested but only two seed concentrations (0.5 ng and 50 pg) shown here for brevity. Cerebellar slice cultures were prepared from *Tga*20 mice. Abbreviations: p1 = passage 1, WT slices treated with brain homogenate; p2 = passage 2, infected slice cultures harvested at 35 dpi, homogenized and used to treat fresh WT slice cultures). (**B**) Corticostriatal slice cultures were prepared from 11-day-old *Tga*20 pups in similar fashion as cerebellar slice cultures and maintained for 42 dpi before testing on RT-QuIC assay. The seeding activity from RT-QuIC traces demonstrates that the competence of seeding activity is highly dynamic and similar to seeds obtained from organotypic cerebellar slices.

To assess the infectivity of the prions generated in slice cultures, we tested the ability of homogenates prepared from RML scrapie-infected brain slice cultures (P1) to infect cerebellar slices (P2) that had been cultured uninfected in parallel with the infected batch. Slices were cultured for 30 days with log dilutions ranging from 0.5 ng–50 fg before testing via RT-QuIC assay. Both P1 and P2 slice cultures demonstrated comparable seeding activity at the dilutions shown (Fig. 6A). The use of cerebellar regions alone required a large number of pups for the study. Thus, to expand the number of slice cultures prepared from a given number of pups, we next tested whether other brain regions can be adopted for an organotypic culture model of prion infection. We infected and cultured non-cerebellar brain sections (corticostriatal slices) for 42 days before testing for seeding activity. To our surprise, these slices demonstrated excellent seeding congruent with the seeding activity obtained from cerebellar slice cultures (Fig. 6B), with seeded amplification occurring in a concentration-dependent manner across a high dynamic range (50 ng to 50 fg) of seed concentrations. Together, these results show the versatility and utility of OSCAR as a rapid bioassay for prion diseases.

## Discussion

We developed a versatile system for quantifying prion seeding activity known as OSCAR that uses the RT-QuIC assay to detect prions in organotypic slice cultures. It is imperative to develop such integrated models for drug development against chronic neurodegenerative conditions like prions and related protein misfolding diseases that can remain asymptomatic for several years. The OSCAR model could advance the utility of the prion organotypic slice culture assay (POSCA) for this infectious disease^26^. However, the caveat for both approaches is that they require adequate technical expertise to prepare and long-term maintenance of the cultures. Nevertheless, we demonstrate the efficacy of prion-infected organotypic slice cultures coupled with seeding assays like RT-QuIC to quantify seeding prions generated in the cultures in a more cost-effective way. Among various *in vitro* culture models, the major advantage of organotypic slice culture is that it preserves morphologic architecture and physiologic interconnectivity between various cell types like neurons and neuroglia. In addition, chronic animal distress associated with *in vivo* prion infection would be circumvented while permitting continuous access to brain tissue for experimental procedures throughout the course of infection. The slices were maintained in culture for several weeks with good viability. Slices infected with scrapie accumulated PK-resistant prions in culture while demonstrating neurotoxicity (Fig. 1A–G) consistent with previous reports^6^,^12^,^24^,^25^. We found the RT-QuIC assay amplifies prions from minute quantities of protein extracted from slice cultures in both a seed concentration- and culture time-dependent manner (Fig. 3A–E) and can be quantified based on seeding characteristics (Fig. 4A,B). Importantly, prion knockout slices infected with RML scrapie did not show any seeding activity across a wide range of seed dilutions (Fig. 2C). The level of seeding activity sustained in our WT slice cultures increased with the duration of the culture period and is comparable to published values^12^. Indeed, the seeding activity that we detected as early as 7 dpi was unexpected, suggesting that the OSCAR model is more sensitive than the prevailing methods of detecting infectivity such as Western blots. This rapid amplification is relevant to recent discoveries that the replication of PrP^Sc^ was observed in vasculature very early after microinjecting prions into mouse brains^27^. Further more, the inoculum used was not detectable in 1-dpi slices possibly due to 1) the washing steps involved in culturing the slices and 2) the homogenized slices were further diluted before testing.

We demonstrated the sensitivity of detecting prions with our OSCAR model of *ex vivo* prion infection and characterized the kinetics of prion amplification at various time points in culture (Fig. 3). The time course of prion amplification in slice cultures is analogous to the kinetic pattern of *in vivo* bioassays as PK-resistant prions rapidly reach maximum titer towards the terminal stage of infection, which becomes accelerated in the *ex vivo* preparation. Based on multiple endpoint titrations for RML scrapie prions in slice culture, seeding activity shows a progressive and time-dependent amplification. The SD_50_ values derived from the Spearman-Kärber method serve as reliable estimates of prion titers from infected slice cultures, indicating that after 35 dpi prion titers are comparable to terminally infected brains^23^. Detection of prions by traditional methods depends on various factors such as PK concentration, incubation time, and choice of antibody that could create discrepancies in the results obtained between samples. Since RT-QuIC seeding activity is not solely based on protease resistance^28^, the OSCAR model could likely be adapted to other seeding prion species. However, it remains to be tested whether this model can be used successfully for other strains and species. As the detection strategies continue to improve for a broad range of prions and strains^28^,^29^,^30^,^31^, we believe that our OSCAR model could be useful in developing procedures that disrupt prion propagation. Distinct kinetic traces from an OSCAR assay could help us to understand more characteristics of prion strains. Finally, using recombinant bank vole (*Myodes glareolus*) PrP as a substrate, a variety of prion strains could be rapidly amplified in an RT-QuIC assay to better understand mechanisms and alternative therapeutic approaches^29^.

This OSCAR model offers a high-throughput platform for extending the utility of integrated prion models to support rapid drug discovery efforts and antemortem diagnostics. As proof-of-principle, we used the OSCAR system to test a small panel of anti-prion compounds that were characterized in animal models of prion diseases^11^,^32^,^33^. First, we used the OSCAR model to test Congo red for its potent curing effects in prion-infected cells^32^. The mechanism of action of this polyanion is its high-binding affinity to amyloids that hyperstabilizes the aggregates^34^, thus interfering with the formation of toxic oligomers. Although Congo red is neuroprotective in prion-infected cerebellar slices^35^, a paradoxical increase in PK-resistant prions, as shown with stronger immunoblotted band intensity, has also been reported^25^. Interestingly, using the OSCAR model, we found it reduced the seeding activity in RML scrapie-infected slice cultures (Fig. 5A–E), thereby supporting a neuroprotective role. Next, we evaluated the small molecule quinacrine. Previous reports suggest treatment with quinacrine results in a strong anti-prion effect in cell culture and it is routinely used in screening libraries^36^,^37^. However, efforts to translate this compound in human trials failed^38^. As further studies determined that quinacrine exhibits poor CNS bioavailability, attempts were made to increase its bioavailability using mice deficient in the P-glycoprotein multi-drug resistance (MDR) transporter. However, this study showed only a transient reduction in both PrP^Sc^ and its conformational stability while encouraging drug resistance^39^. In slice cultures, chronic treatment with quinacrine showed an intermediate decrease in seeding activity when compared to control slices infected with RML scrapie (Fig. 5A–E). This anti-prion effect in cultured slices could be attributed to direct interaction of the drug with the brain slices and early initiation of the treatment regimen. Lastly, we tested astemizole, as it was reported to show anti-prion activity in cell culture models and to marginally extend the survival of prion-infected mice (~4%) despite having no effect on reducing PrP^C^ levels or PrP mRNA levels in PK1 cells^11^. Astemizole crosses the blood-brain barrier and reduced the PK-resistant PrP in the persistently infected cell culture model. Although its exact anti-prion mechanism of action is not known, it has been shown to stimulate autophagy. Conversely, in our study, astemizole did not show any reduction in seeding activity and the seeding kinetics remained similar to that of cerebellar slice cultures infected with RML scrapie (Fig. 5A–E). This difference may be attributed to a highly sensitive readout using RT-QuIC assay to detect seeding prions. These initial studies demonstrate the translational potential for the OSCAR model to further test novel compounds and may have utility in future treatment paradigms involving a wide range of prion strains.

Emerging evidence suggests that protein misfolding plays a key role in major neurodegenerative disorders that have prion-like templated conversion of proteins, including Parkinson’s, Alzheimer’s, frontotemporal dementias, multiple sclerosis, multiple system atrophy and Huntington’s diseases. No drugs are available for halting or reversing prion diseases or any other prion-like neurodegenerative disorders^40^. Additionally, drugs demonstrating excellent effects in cell culture and *in vivo* models of scrapie paradoxically fail to show any effect on CJD prions^15^ or in clinical trials^38^. Also, mouse cell lines persistently infected with prions were developed to establish *in vitro* models of scrapie^41^,^42^ for drug testing, but compounds identified through a high-throughput drug screening^43^ had little or no effect against CJD prions. Furthermore, no cell culture model can propagate the CJD prions in culture. We speculate that the OSCAR model could be extended to CJD human prions, thereby filling a critical void because currently available models are medically intractable against CJD prions.

**Figure 7:**
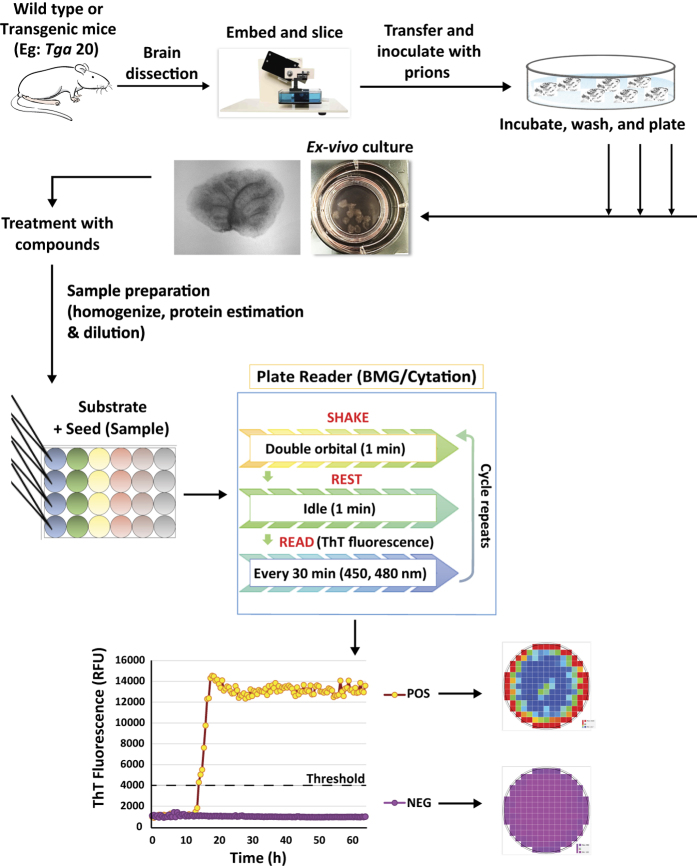
Schematic illustration of OSCAR workflow. The brain slice cultures were prepared in organotypic fashion obtained from neonatal mice and embedded in compressing lip and sliced. The slices were incubated with either normal or prion-infected brain homogenates for 1 h, then washed and cultured with liquid air interphase on membrane inserts. Treatments with compounds of interest began at 14 dpi in this study and slices were harvested at 31 dpi. Slices were homogenized and dilutions were used to seed RT-QuIC reactions. RT-QuIC assay was performed in 96-well plate using recombinant prion protein as a substrate with continuous shake/rest cycles. The seeding activity was monitored in real time based on seeding using a thioflavin T (ThT) fluorescence readout and quantified. Positive wells can be clearly visualized based on the traces that cross the threshold fluorescence and by the rapid increase in intensity. The well-area scan image shows the intense amyloid deposits in the well.

In summary, we have established a highly sensitive integrated technique for the efficient detection of seeding prions (Fig. 7). Our OSCAR model using an organotypic slice culture assay coupled to the RT-QuIC assay may be able to accelerate translational research by testing a large number of anti-prion compounds, including small molecules, against prion infection. Furthermore, the OSCAR model could act as a prototype for other protein misfolding disorders (PMDs) that obey principles of aggregation and propagation. In addition, using OSCAR with transgenic mouse models and genetic manipulation may provide mechanistic insights into the cell-to-cell transmission of prion-like aggregates and the progression of neurodegenerative diseases.

## Materials and Methods

### Reagents

Dithiothreitol (DTT), ethylenediaminetetraacetic acid (EDTA), 45% glucose, kynurenic acid, proteinase K (PK), sodium chloride (NaCl), Tris-HCl and Triton X-100 were purchased from Sigma (St. Louis, MO). Millicell 6-well plate inserts (Biopore filter CM, PTFE inserts 30-mm diameter and 0.4-um pore size, Cat No. PICM03050), *E. coli* Rosetta DE3 competent cells, Overnight Express^TM^ Autoinduction System 1 (Cat. no. 71300-3) and BugBuster^®^ Master Mix (Cat No. 71456-4) were purchased from Millipore (Billerica, MA). Bradford protein assay kit was purchased from Bio-Rad Laboratories (Hercules, CA). Horse serum, penicillin, streptomycin, and low-melting agarose (Cat No. 15517-022) were purchased from Invitrogen (Carlsbad, CA). Anti-PrP mouse monoclonal antibodies POM1 and 6D11 were purchased from Prionatis AG (Schlieren, Switzerland) and Covance, respectively. Anti-β-actin antibody was purchased from Sigma-Aldrich. Alexa Fluor 680 conjugated goat anti-mouse IgG was purchased from Invitrogen. Goat anti-rabbit IR800 conjugated IgG was purchased from Rockland Immunochemicals (Gilbertsville, PA), propidium iodide (PI) from Molecular Probes (Cat No. P3566), and Halt™ Protease and Phosphatase Inhibitor Cocktail (Cat No. 78440) from Life Technologies. All other reagents were obtained from Thermo Fisher Scientific (Waltham, MA) unless otherwise mentioned.

### Slice cultures and infections

All the experiments involving animals followed protocols approved by Iowa State University’s Institutional Animal Care and Use Committee (IACUC) and by IACUC at the National Animal Disease Center (protocol number: 3985). Organotypic cerebellar slices for prion assay were prepared as previously described^6^,^26^. In brief, brain slices were prepared from 9- to 12-day-old mouse pups from either wild-type (WT, C57BL/6) or transgenic prion-overexpressing or knockout mice (*Tga*20 or prnp^−/−^, respectively) using a microtome (Compresstome™ VF-300, Precisionary Instruments). After dissecting the cerebellum from the whole brain, the cerebellum was oriented in the sagittal plane in the Compresstome’s specimen tube, which had been prefilled with 2% low-melting-point agarose. The agar was quickly solidified by clasping the specimen tube with a chilling block, and then the specimen tube was inserted into the slicing reservoir filled with freshly prepared, ice-cold Gey’s balanced salt solution supplemented with the excitotoxic antagonist, kynurenic acid (GBSSK). To prepare GBSS, we added the following in solution in the following order from 10x stocks to obtain the final concentrations per liter: 8 g NaCl, 0.37 g KCl, 0.12 g Na_2_HPO_4_, 0.22 g CaCl_2_ ∙ 2H_2_O, 0.09 g KH_2_PO_4_, 0.07 g MgSO_4_ ∙ 7H_2_O, 0.210 g MgCl_2_ ∙ 6H_2_O, 0.227 g NaHCO_3_. The compression lip located in the cutting chamber helps stabilize the brain specimen while obtaining 350-μm thick slices with the blade set at a medium vibration speed. Slices were collected at the specimen tube’s outlet and transferred to another plate with fresh prefilled GBSSK. Slices were exposed on ice for 1 h to either normal brain homogenate (NBH) or Rocky Mountain Labs scrapie brain homogenate (RML scrapie) with 100 μg/ml of brain homogenate total mass diluted in 1 mL GBSSK. Later, the slices were washed twice in 6 ml ice-cold GBSSK, transferred to Millicell 6-well plate inserts (7–9 slices per insert) and were incubated in a humidified atmosphere of 5% CO_2_ at 37 °C. Culture media was exchanged every other day with fresh media until the infected slices reached their pre-determined endpoints. For scaling up the experiments, we cultured a pool of ~50 slices per 6-well plate, all from a single litter with the same genotype, and exposed to either NBH or RML scrapie brain homogenates. These slices were triple-washed in ice-cold GBSSK before plating and maintained as described above. Slice cultures were harvested at various endpoints by washing twice in 2 ml of ice-cold PBS. Lysates were prepared in 1X PBS by three freeze-thaw cycles and subjected to five 30-sec sonication pulses each in a cup sonicator. When testing anti-prion compounds, the compounds were added from stock solutions (1000X) to slices at 15 days post-inoculation (dpi), and then fresh media with compounds was exchanged every other day and harvested at 31 dpi as described above.

### Viability assays

Viability assays in organotypic cerebellar slices were performed as previously described with modifications^44^. Dead cells were selectively stained by incubating the slices in culture media with 5 μg/ml of PI, which is a nontoxic dye that selectively binds to nuclei of dead cells and can be used to image live slice cultures. After 30 min, the PI media was replaced with fresh culture media before imaging at 2X and 10X magnification through a Nikon TE2000-U microscope (Tokyo, Japan) coupled to a SPOT digital camera (Diagnostic Instruments, Sterling Heights, MI). All the images were processed using ImageJ with constant threshold settings.

### Immunoblotting

Western blot analyses and limited proteolysis were performed as described previously^6^,^7^,^25^,^45^ with minor modifications. Briefly, slices were washed twice in ice-cold PBS and harvested with PBS, unless otherwise specified. After protein assay, equal homogenates were separated on 15% SDS-PAGE gels. After electrophoresis, proteins were transferred to a nitrocellulose membrane. The membranes were blocked using LI-COR blocking buffer (LBB) for 1 h and probed overnight with the primary antibody followed by secondary antibody treatments using Alexa Fluor-conjugated anti-mouse or anti-rabbit antibodies. An Odyssey IR Imaging (LI-COR) system was used to capture and analyze images using Odyssey 2.0 software, and densitometry was done using ImageJ. For limited proteolysis, we used partial Proteinase-K digestion methods as previously described with a few modifications^6^,^12^,^13^. At 35 dpi, RML scrapie- and NBH-inoculated slice cultures were homogenized in lysis buffer (1% Triton-X in PBS). After determining the protein concentration by Bradford assay, 200 μg of protein was digested with 25 μg/ml of Proteinase-K and incubated for 30 min at 37 °C. When brain homogenates were used, 100 μg of protein was digested with 25 μg/ml of Proteinase-K for 45 min at 37 °C. The digestion reactions were stopped by boiling the samples for 10 min. Samples were stored at −80 °C until Western blot analyses were performed as described above. Membranes were incubated with the POM1 PrP antibody (1:5000, Prionatis AG) overnight at 4 °C and then triple-washed with 0.1% Tween in 1X PBS. Secondary antibody incubations and washings were done as described above and fluorescence was captured using the Odyssey IR Imaging system.

### Dot Blot

Immunoreactivity of PrP from the prion-infected slice cultures was determined by dot-blot using Bio-Dot^®^ Microfiltration System per manufacturer’s protocol as described previously^46^. In brief, the slice cultures were harvested at pre-determined endpoints, and homogenates were prepared and protein concentrations were determined as described above. Next, 0.5 μg and 5 μg of homogenates were mixed with 200 μl of tris-buffered saline (1X TBS) with 0.1% Tween-20 and allowed to adsorb onto a nitrocellulose membrane for 1 h. The membrane was washed twice with 200 μl of 1X TBS using a gentle vacuum, blocked with 1X LBB for 30 min, incubated with mouse monoclonal POM1 anti-PrP antibody (dilution 1:5000) for 1 h at RT, and triple-washed with 1X TBS. Membranes were then incubated with Alexa Fluor 680-conjugated anti-mouse secondary antibody in LBB (1:10,000) for 30 min followed by 3 washes in 1X TBS. As the loading control, membranes were probed with β-actin. Antibody-bound proteins were detected with the Odyssey IR Imaging system and densitometry was performed with ImageJ.

### Immunohistochemistry

After fixing the slices, antibody treatments were performed as described previously^6^,^25^,^47^ with minor changes. Slices were rinsed twice with 1 ml of 1X PBS and fixed in 4% paraformaldehyde (PFA) overnight at 4 °C. Before antibody treatments, slices were washed twice with PBS for 15 min each at RT to remove residual PFA and incubated with blocking buffer (0.05% TritonX-100 and 3% goat serum in 1X PBS) for 1 h at RT. Primary antibodies were diluted (1:1000 unless otherwise stated) in blocking buffer and incubated for 2–3 days at 4 °C. The following antibodies were used: anti-NeuN (clone A60, Alexa Fluor^®^488 conjugated Millipore, Cat No. MAB377X) and anti-PKCδ (Santa Cruz). After incubation with primary antibody, membranes were washed 4 times with PBS for 15 min each and incubated 1 h in the dark with the following secondary antibodies diluted 1:1500 in blocking buffer: Alexa Fluor 555-conjugated anti-mouse secondary antibody or Alexa Fluor 488-conjugated anti-rabbit secondary antibody. The cell-permeable dye Hoechst 44432 (1:5000 for 3 min in PBS) was used to stain all nuclei. The culture membranes were removed from the inserts and mounted directly on microscope slides, with membranes facing the slide, using Fluoromount mounting medium (Sigma) and imaged with a SPOT color digital camera attached to a Nikon TE2000-U microscope.

### Recombinant prion protein expression and purification

Recombinant prion protein (rPrP) was expressed and purified using previously reported protocols^23^,^28^,^48^,^49^,^50^. Briefly, N-histidine-tagged prion protein-encoding plasmids of Syrian golden hamster residues for full length (23–231) or truncated (90–231) in pET vector (EMD biosciences) were transformed into *E. coli* Rosetta^TM^2 (DE3) cells and grown under kanamycin and chloramphenicol as selection media. Protein expression was induced using Overnight Express™ Autoinduction System 1 (EMD Millipore Cat No. 71300) with log phase mini-cultures as a starter culture. The bacteria were pelleted and lysed with BugBuster^®^ Master Mix to isolate inclusion bodies. Inclusion bodies were dissolved in 8 M of the denaturing agent guanidine hydrochloride (GuHCl) for 1 h and mixed to pre-equilibrated nickel-nitrilotriacetic acid (Ni-NTA Superflow) resin (Qiagen Cat No. 30430) for 50 min on a rotating mixer. The beads were packed in an Akta #XK26 column and purified using Ni-NTA-immobilized metal affinity chromatography (IMAC) on a Bio-Rad DuoFlow system operating at 4 °C. On-column refolding was performed using gradient reduction of GuHCl. Pure rPrP was eluted from the IMAC using competitive imidazole binding with a linear gradient. Chilled dialysis buffer was added to the peak eluted fractions, filtered and dialyzed against three changes of chilled dialysis buffer (10 mM NaPO_4_, pH 5.8). Following dialysis, the rPrP was filtered, aliquoted and stored at −80 °C. Protein concentration was determined by absorbance at 280 nm over the extinction coefficient before freezing as well as after thawing and filtering through 100-kD cutoff filters. Protein concentration was typically in the range of 0.5–0.7 mg/ml after dialysis. Each batch was routinely tested for quality and activity using Western blot and RT-QuIC assay respectively, before using on test samples.

### RT-QuIC assay

RT-QuIC assay was performed using standard protocols from published reports^23^,^30^,^51^ with slight modifications. Unless otherwise specified, the reaction mixtures consisted of final concentrations of 350 mM NaCl, 0.1 mM EDTA, 10 μM ThT, 0.1 mg/ml rPrP and 0.0025% SDS in 1X PBS. First, 5-μL samples from either brain homogenates or slice culture homogenates were diluted in a total reaction mixture of 100 μL. Plates subjected to RT-QuIC assay were sealed with Nalgene Nunc plate sealer. Plates were incubated at 42 °C in either a CLARIOstar (BMG) or Cytation3 (BioTek) plate reader with alternating 1-min shake (double orbital) and rest cycles. All samples were run at least in triplicates, and samples were judged to be positive as reported previously when samples were run in quadruplicates^30^,^50^. Whenever triplicates were run, we averaged their fluorescence readings, and additionally we selected 10xSD (standard deviation of negative controls) as the criteria for determining the threshold. Bottom plate recordings of ThT fluorescence (450 ± 15 nm excitation and 480 ± 10 nm emission) were taken every 30 min and data analysis was performed as reported previously^16^ using MARS version 5.2.R8 or Gen 5 version 2.07.17 software when CLARIOstar (BMG) or Cytation3 (BioTek) were used, respectively, and the data were exported to Microsoft Excel for graphing.

## Additional Information

**How to cite this article:** Kondru, N. *et al*. Integrated Organotypic Slice Cultures and RT-QuIC (OSCAR) Assay: Implications for Translational Discovery in Protein Misfolding Diseases. *Sci. Rep.* **7**, 43155; doi: 10.1038/srep43155 (2017).

**Publisher's note:** Springer Nature remains neutral with regard to jurisdictional claims in published maps and institutional affiliations.

## Supplementary Material

Supplementary Information
