# Supplementary material for: Integrated Organotypic Slice Cultures and RT-QuIC (OSCAR) Assay: Implications for Translational Discovery in Protein Misfolding Diseases
Source: Sci Rep. 2017 Feb 24;7:43155. doi: 10.1038/srep43155 (PMC5324099; doi:10.1038/srep43155)
Supplement: Supplementary Information [file srep43155-s1.pdf]

Supplementary materials

Integrated Organotypic Slice Cultures and RT-QuIC (OSCAR) Assay: Implications for Translational Discovery in Protein Misfolding Diseases

Naveen Kondru[1], Sireesha Manne[1], Justin Greenlee[2], Heather Greenlee[1], Vellareddy Anantharam[1], Patrick Halbur[3], Arthi Kanthasamy[1], Anumantha Kanthasamy[1*]

1. Department of Biomedical Sciences, College of Veterinary Medicine, Iowa State University, Ames, IA 50011, USA.
2. Virus and Prion Research Unit, National Animal Disease Center, Agricultural Research Service, United States Department of Agriculture, Ames, Iowa, USA.
3. Veterinary Diagnostic and Production Animal Medicine, College of Veterinary Medicine, Iowa State University, Ames, IA 50011.

*** Corresponding author:** akanthas@iastate.edu

Supplementary Figure 1

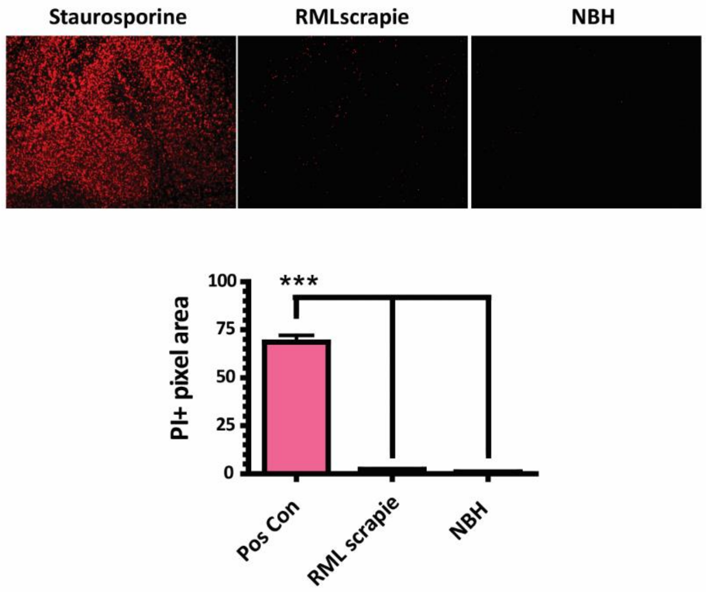

Supplementary Fig 1. Organotypic slices are viable for a long period of time in culture. Slices cultured for 63 days were assessed for viability using Propidium iodide (PI) fluorescence. Percentage of relative PI uptake intensity was used to compare cell death. Staurosporine evokes significantly stronger cell death as compared with the slices cultured post infection with either NBH or RML scrapie.

Supplementary Figure 2

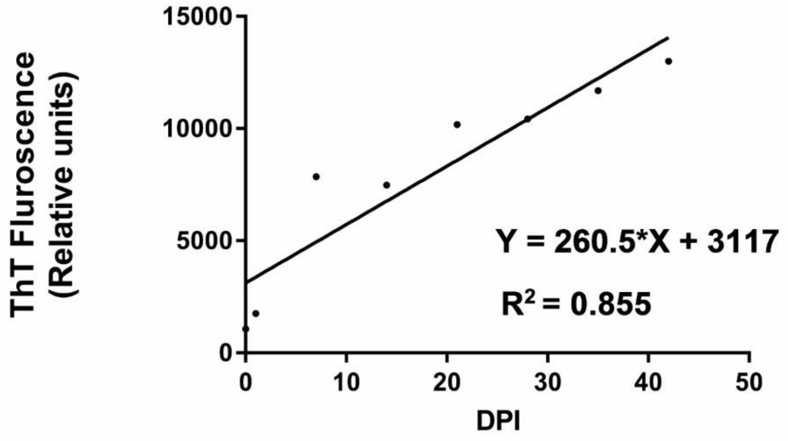

Supplementary Fig 2. Slice cultures illustrate the time-dependent increase in average fluorescence readings. Correlation between days post infection (DPI) versus an increase in the average fluorescence readings from the seeded reactions.

Supplementary Figure 3

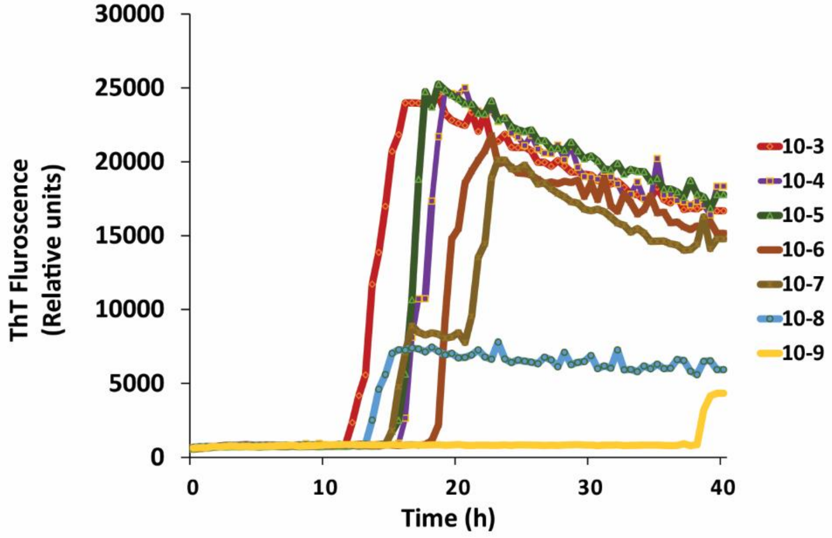

Supplementary Fig 3. Determining prion seeding activity from end point dilutions of infected scrapie brain homogenates. Serial dilutions of RML scrapie brain homogenates were used to seed RT-QuIC reactions; average of triplicate traces are shown. The 50% seeding dose (SD_{50}) was calculated based on the number of wells turned positive for each dilution, while the SD_{50} was calculated as described in methods. In this case, approximate SD_{50} for the RML scrapie brain homogenate was achieved with 5 ul seed from a 10^{-8} dilution.

Supplementary Figure 4

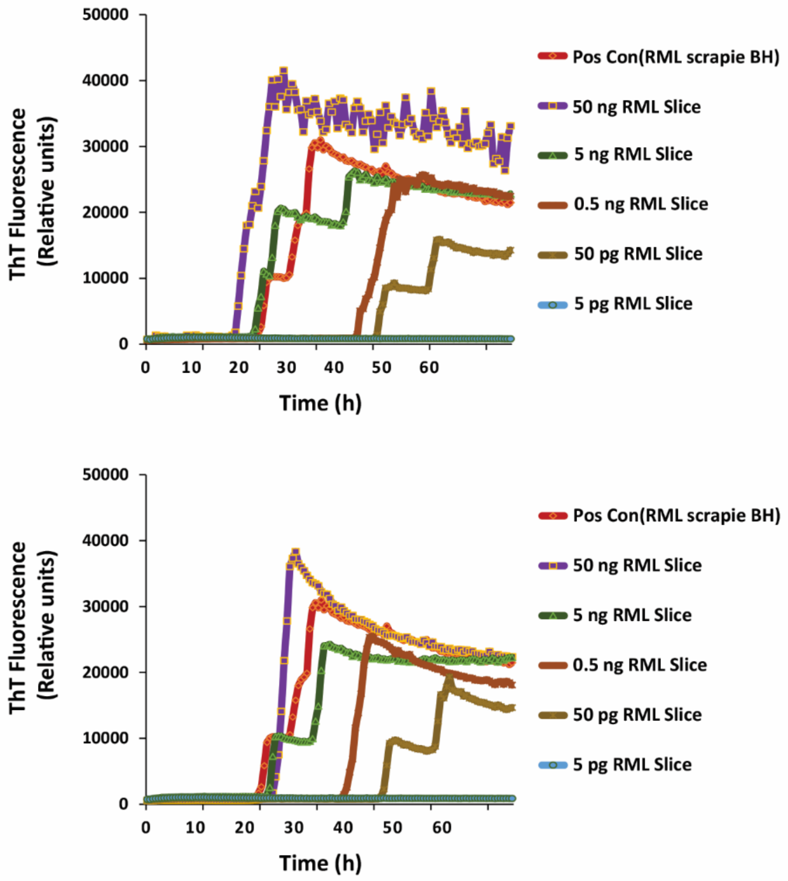

Supplementary Fig 4. Prion seeding kinetics between biological replicates have high reproducibility. RT-QuIC reactions seeded with the slice cultures from two separate experiments show similar seeding kinetics across the wide range of dilutions tested.

Supplementary Fig 5

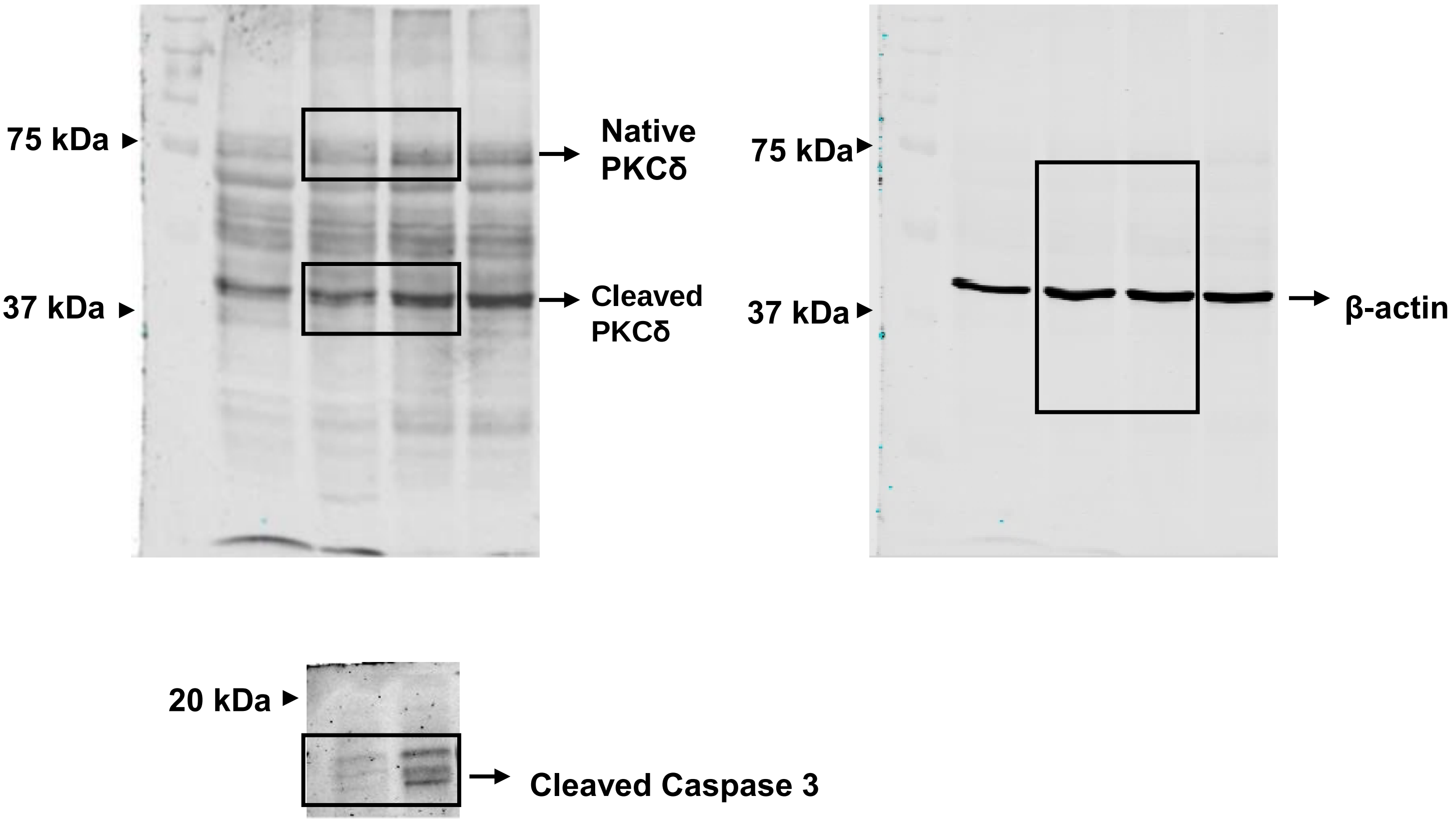

Supplementary Fig 5. Original western blots for the cropped images used in Fig 1G. Regions of western Blots shown in the figures 1G were highlighted in black boxes.
